# Suicidality in the Arab World: Results from an Online Screener

**DOI:** 10.1007/s10597-023-01129-7

**Published:** 2023-05-04

**Authors:** Sariah Daouk, Mina Dailami, Suzanne Barakat, Rania Awaad, Ricardo F. Muñoz, Yan Leykin

**Affiliations:** 1 Department of Psychology, Palo Alto University, 1791 Arastradero Rd, Palo AltoPalo Alto, CA 94304 USA; 2 Department of Family & Community Medicine, University of California, San Francisco, San Francisco, CA USA; 3 Department of Psychiatry and Behavioral Sciences, Stanford University, Palo Alto, CA USA; 4 Department of Psychiatry and Behavioral Sciences, University of California, San Francisco, San Francisco, CA USA; 5 Institute for International Internet Interventions for Health, Palo Alto, CA USA

**Keywords:** Arab, Suicide, Suicidality, Depression

## Abstract

Suicide in the Arab World is grossly understudied. This study sought to understand suicidality among Arabic-speaking individuals visiting an online depression screener. A large sample (N = 23,201) from the Arab World was recruited online. 78.9% (n = 17,042) reported suicidality (thoughts of death or suicide, or a suicide attempt) and 12.4% reported a suicide attempt in the past 2 weeks. Binary logistic regressions indicated that women tended to report more suicidality, and that suicidality tended to decline with age (all *p*s < 0.001), across all levels of suicidality. Comparing countries with n ≥ 1000 (Algeria, Egypt, Jordan, Morocco, and Saudi Arabia), several 3-way (gender * age * country) and 2-way interactions indicated that some countries departed from the usual pattern of responses. For instance, in Algeria, neither gender nor age differences were observed in reported attempts. Women and younger adults in the Arab World may be at higher risk of suicidality. Differences between and within countries warrant further exploration.

## Introduction

The World Health Organization estimates that 700,000 people die by suicide every year, and the number of attempts is estimated to be considerably larger (SAMHSA, 2018; World Health Organization, 2019). In the 22 countries comprising the Arab World, suicide and suicidality (which for the purposes of this study is defined as any ideation such as thoughts of death or suicide, or actual suicide attempts) are largely unexplored (Daouk et al., 2020). Large-scale studies estimate that suicide rates in the Arab World are low (3.9 to 4.8 per 100,000) compared to average global rates (9.0 per 100,000) (Naghavi, 2019; World Health Organization, 2019). However, the subject of suicide – and mental illness overall – is generally regarded as a taboo in the Arab World, and as a result, suicidality is highly underreported and very likely grossly underestimated. For instance, recent research suggests that individuals in the Arab World are reporting high rates of depressive symptoms and suicidal ideation (Fayyaz & Beg, 2019).

Though suicide prevalence rates in the Arab World may vary by country (Naghavi, 2019), there appear to be certain commonalities based on individuals’ age. Rates of death by suicide are highest for people under the age of 30, comprising 38.6% of all deaths by suicide (Naghavi, 2019), with the greatest prevalence of suicidal behavior among those aged 20–24 (Amini et al., 2021). Indeed, Eskin and colleagues (2019) found that 22% of university students living in predominantly Muslim Mediterranean countries reported suicidal ideation and 8.6% reported attempting suicide. Alarmingly, some studies suggest that suicidality may occur even earlier, with 28.9% of Lebanese adolescents reporting suicidal ideation (Chahine et al., 2020). Beyond young adulthood, suicide rates decrease with age, and then increase again for people over age 65 (Amini et al., 2021).

In addition to variability by age, suicidality in the Arab World appears to vary across genders, in a manner consistent with global trends (Värnik, 2012). There is a higher prevalence of death by suicide for men than for women in the Arab World, but a higher prevalence for suicide ideation, planning, and attempts for women than men (Eskin et al., 2019; Halabi et al., 2020). In Iraq and Syria, for example, between 2011 and 2012, 78% of adults coming to the emergency room for suicide attempts were women (Halabi et al., 2020). Lethality of attempts appear to differ as well, with fewer suicide attempts made by women requiring medical attention when compared to men (Halabi et al., 2020).

The Internet has been a valuable source of screening, treatment, and education for various mental health concerns, including depression and suicidality (Büscher et al., 2020; Houston et al., 2001; Sin et al., 2020; Tate & Zabinski, 2004). The value of Internet resources extends to individuals who are low-income or less educated or those residing in low- and middle-income countries (Brodie et al., 2000; Fu et al., 2020). Additionally, health information can be obtained on the Internet in an anonymous, private, immediate, and convenient manner (Bischoff & Kelley, 1999; Skinner et al., 1982). The relative privacy of the online space may help individuals disclose their suicidality more openly, including in those areas of the world where mental health and suicidality are considered socially stigmatizing or unacceptable. Indeed, though stigma regarding mental illness and suicidality for individuals in the Arab World presents a barrier to seeking in-person care, Internet-based psychological services may be more acceptable (Househ et al., 2019; Hughes et al., 2020; Ibrahim et al., 2019).

Additionally, since the beginning of the COVID-19 pandemic, Internet use throughout the region has increased considerably across multiple domains, including for content related to health (Allagui, 2021; Kamel et al., 2020; Kayrouz et al., 2018). Internet-based psychological assessments may therefore be both acceptable and effective way of gaining insight into the prevalence of suicidal ideation and suicide attempts among those seeking help online in this region. The goal of the study is therefore to understand the prevalence of suicidality in different demographic populations in the Arab World in a sample of individuals who took part in a free online depression and suicidality screener.

## Methods

### Participants

Participants were recruited to an online depression and suicidality screener (Leykin et al., 2012) primarily via Google Ads, formerly Google AdWords (Gross et al., 2014); other participants may have found the study through links from other websites or word of mouth. The screener is available in five languages; given the purposes of this investigation, only the Arabic-speaking sample is considered here. Thus, participants in this study were 23,201 individuals who were 18 + years of age, Arabic-literate, and living in one of the 22 Arab countries. Only participants who identified as men or women were included in the analyses (263 who identified as Other and 1167 who preferred not to state their gender were not included in this analysis). Demographic information for the sample can be found in Table 1.

**Table 1:** Characteristics of the Sample

	OVERALL (N = 23,201) % or *M*(*SD*)	Egypt (n = 11,229) % or *m*(*SD*)	Saudi Arabia (n = 3,064) % or *m*(*SD*)	Morocco (n = 1,910) % or *m*(*SD*)	Jordan (n = 1,599) % or *m*(*SD*)	Algeria (n = 1,268) % or *m*(*SD*)	Comparing 5 Countries *p*-value
Age	26.84 (8.78)	26.40 (8.45)	27.18 (9.12)	26.74 (8.77)	25.78 (9.27)	27.58 (7.94)	< .001
% Female	57.1%	53.8%	54.5%	68%	60.6%	64.5%	< .001
Current Suicidality	78.9%	82.7%	77.5%	72.7%	79.7%	68.5%	< .001

*Note: p*-values are based on an ANOVA (age) and chi-square tests (gender and suicidality)

### Measures

#### Demographics

Participants reported their age, gender, and country of residence.

#### The Major Depressive Episode (MDE) Screener

The MDE Screener is an 18-item screener based on the Diagnostic Interview Schedule (Muñoz, 1998; Robins et al., 1981) that assesses for the presence of Current and Past major depressive episodes (Muñoz et al., 1999; Vázquez et al., 2008). The instrument has been translated into Modern Standard Arabic (Daouk et al., 2020). The following Yes/No items from the MDE screener were used to define Current (past 2 weeks) suicidality:

- Thought a lot about death—either your own, someone else’s, or death in general?
- Wanted to die?
- Felt so low you thought about committing suicide?
- Attempted suicide?

### Procedure

Study procedures are detailed elsewhere (Leykin et al, 2012). Briefly, participants conducting Google searches in Arabic for terms related to depression, suicide, or mood may have seen one of the ads for an online mood or depression screener. Those clicking on an ad proceeded to a page describing the study in more detail; the page also included a statement that data will be retained for research purposes. Participants then completed demographic questions; those under the age of 18 (Stephens et al., 2020) were informed of their ineligibility and provided resources about depression. Adult participants were then directed to the MDE screener, reported their current depression symptoms, and were offered feedback on their results. Participants were also asked whether their answers were “accurate” or whether they were “just testing” the website (those indicating the latter were excluded from the analyses). Participants reporting suicidality were shown a screen expressing concern and urging them to reach out to local and online resources. Participants were then invited to take part in a monthly depression re-screening study. Those consenting to this study completed additional measures (not discussed here) and were sent monthly invitations to re-screen themselves for depression, for 12 months. Study procedures were approved by the Institutional Review Boards at the University of California, San Francisco and Palo Alto University.

### Statistical Approaches

To identify factors related to different levels of suicidality, binary logistic regression models were used. Participants were mainly younger, therefore age was recategorized into four groups: 18–21, 22–29, 30–39, and 40 and above. Age groups, gender (man or woman; those indicating “other,” or preferring not to disclose their gender were excluded from analyses), and age by gender interactions were used as predictors in logistic regression models, with levels of suicidality (thoughts about death; wanting to die; thoughts of suicide; and past suicide attempt) as dependent variables. First, four binary logistic regression models were used to understand the association of demographic characteristics with each level of suicidality for the entire sample. Second, to understand whether demographic characteristics differ for the five countries with the highest number of participants (Algeria, Egypt, Jordan, Morocco, and Saudi Arabia), these models were repeated, with the addition of the country as a predictor and including two-way and three-way interactions of country and predictors; non-significant interactions were iteratively removed until none remained.

## Results

### Characteristics of the Sample

A total of 23,201 Arabic-speaking adults residing in Arab countries reported answering the questions “accurately.” 21,599 adult participants responded to suicidality items, with 17,042 (78.9%) reporting at least some level of current suicidality. On average, participants were 26.84 years old (SD = 8.78). Categorization of age yielded the following age groups: 18–21 (n = 7,983); 22–29 (n = 8,500); 30–39 (n = 4,422); 40 + (n = 2,296). Regarding gender, 57.1% of participants identified as women (see Table 1). Across the whole sample, 73.0% of participants reported thinking lot about death in the past 2 weeks, 52.7% reported wanting to die, 36.3% reported feeling so low they thought about suicide, and 12.4% reported attempting suicide.

### Full Sample: Relationship of Demographic Characteristics to Suicidality

#### Thoughts of Death

The 2-way gender * age groups interaction was not significant in predicting thoughts of death in the full sample (*Wald chi-square*(3) = 1.30, *p* = 0.73). Regarding main effects, both gender (*Wald chi-square*(1) = 30.65, *p* < 0.001) and age group (*Wald chi-square*(3) = 153.49, p < 0.001) were significantly associated with having thoughts of death. Women’s odds of having thoughts of death were 19.2% higher compared to men (*Wald chi-square*(1) = 30.65, *p* < 0.001, *OR* = 1.19, 95% CI:1.12–1.27). Relative to 18–21 age group, all other age groups had lower odds of having thoughts of death (18.6% lower for 22–29 group: *Wald chi-square*(1) = 29.81, *p* < 0.001, *OR* = 0.81, 95% CI:0.76–0.88; 28.5% lower for 30–39 group: *Wald chi-square*(1) = 57.25, *p* < 0.001, *OR* = 0.72, 95% CI:0.66-0.78; 47.0% lower for 40 + group: *Wald chi-square*(1) = 139.43, *p* < 0.001, *OR* = 0.53, 95% CI:0.48–0.59).

#### Wanting to Die

The 2-way gender * age groups interaction was likewise not significant in predicting wanting to die in the full sample (*Wald chi-square*(3) = 2.07, *p* = 0.56). As above, both gender (*Wald chi-square*(1) = 42.57, *p* < 0.001) and age (*Wald chi-square*(3) = 650.25, *p* < 0.001) were significantly associated with reporting wanting to die. Compared to men, odds of wanting to die were 20.6% higher among women *(Wald chi-square*(1) = 42.57, *p* < 0.001, *OR* = 1.21, 95% CI:1.14–1.28). Similar to above, relative to 18–21 age group, the odds of wanting to die were lower for all other age groups (26.6% lower for 22–29 group: *Wald chi-square*(1) = 87.24, *p* < 0.001, *OR* = 0.74, 95% CI:0.69-0.78; 51.5% lower for 30–39 age group: *Wald chi-square*(1) = 328.04, *p* < 0.001, *OR* = 0.49, 95% CI:0.45–0.52; 69.2% lower for 40 + age group: *Wald chi-square*(1) = 493.14, *p* < 0.001, *OR* = 0.31, 95% CI:0.28–0.34).

#### Thoughts of Suicide

**Fig. 1:**
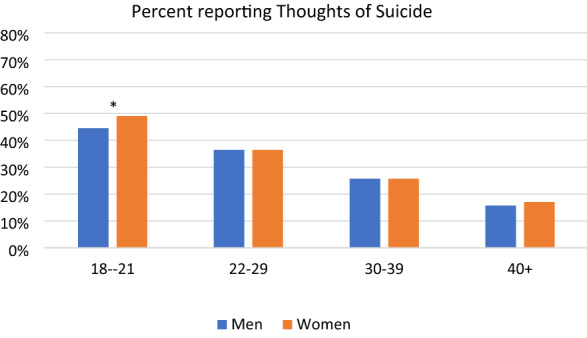
Full Sample: Thoughts of Suicide by Age Groups and Gender *Note*: * = Significant difference based on a Bonferroni-adjusted pairwise z-test for proportions

The 2-way gender * age groups were significantly associated with reporting thoughts of suicide in the full sample (*Wald chi-square*(3) = 7.97, p = 0.047). As can be seen in Fig. 1, relative to men, women in 18–21 age group and in the 40 + age group were more likely to report having thoughts of suicide, whereas for the other age groups, men and women reported thinking about suicide at similar rates.

#### Suicide Attempt

The 2-way gender * age groups interaction was not significant in predicting report of a suicide attempt in the full sample (*Wald chi-square*(3) = 5.12, *p* = 0.16). Similar to findings for thoughts of death and wanting to die, gender (*Wald chi-square*(1) = 152.50, *p* < 0.001) and age (*Wald chi-square*(3) = 452.64, *p* < 0.001) were significantly associated with reporting a suicide attempt in the past 2 weeks. Compared to men, the odds of attempting suicide were 78.8% higher for women (*Wald chi-square*(1) = 152.50, *p* < 0.001, *OR* = 1.79, 95% CI:1.63–1.96). Relative to 18–21 group, the odds of all other groups of making an attempt were lower (47.7% lower for 22–29 group: *Wald chi-square*(1) = 188.07, *p* < 0.001, *OR* = 0.52, 95% CI:0.48–0.57; 67.9% lower for 30–39 group: *Wald chi-square*(1) = 257.83, *p* < 0.001, *OR* = 0.32, 95% CI:0.28-0.37; and 76.7% lower for 40 + group: *Wald chi-square*(1) = 174.19, *p* < 0.001, *OR* = 0.23, 95% CI:0.19–0.29).

## Country-Level Differences in Suicidality: Comparing Five Arab Countries (Fig. 2)

**Fig. 2:**
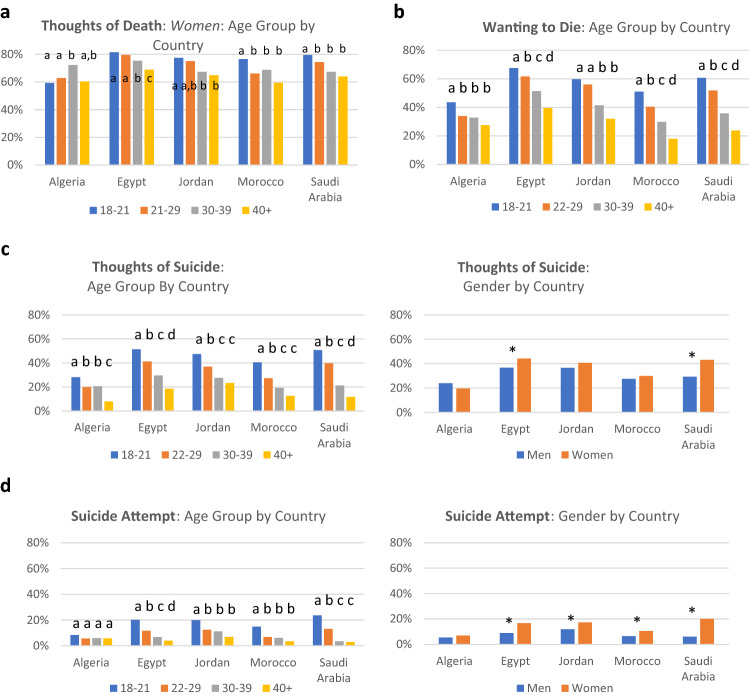
Suicidality Levels in Select Countries *Note*: Letters denote Bonferroni-adjusted pairwise z-tests for proportions, per country, per graph. Different letters denote differences between columns at a .05 level; same letter denotes no significant differences between columns. * = Significant difference based on a Bonferroni-adjusted pairwise z-test for proportions

To determine whether the age- and gender-related differences in suicidality vary between countries, differences between five countries with most sizable representation (n > 1000) in our sample were examined. The countries selected were Egypt (n = 11,229), Saudi Arabia (n = 3064), Morocco (n = 1910), Jordan (n = 1599), and Algeria (n = 1268).

### Thoughts of Death

The 3-way gender * age group * country interaction was significant in predicting thoughts of death in the five countries (*Wald chi-square*(12) = 24.17, *p* = 0.019). To better understand this interaction, men and women were analyzed separately. For men, the 2-way age group * country interaction was not significant (*Wald chi-square*(12) = 19.24, *p* = 0.08). For women, however, the 2-way age group * country interaction was significant (*Wald chi-square*(12) = 29.63, *p* = 0.003). As can be seen from Fig. 2a, the trend of the prevalence of thoughts of death decreasing with increasing age regardless of gender was observed in Egypt, Saudi Arabia, and Jordan. In Algeria, however, 30–39 year-olds were most likely to report thoughts of death.

### Wanting to Die

The 3-way interaction gender * age group * country interaction was not significant (*Wald chi-square*(12) = 17.03, *p* = 0.148). Examining 2-way interactions, the age group * country interaction was significant (*Wald chi-square*(12) = 24.41, *p* = 0.018). As can be seen from Fig. 2b, for Egypt, Algeria, Morocco, and Saudi Arabia, regardless of gender, the likelihood of wanting to die was highest among the 18–21 years old group and declined steeply with each subsequent age group. However, for Algeria, though the 18–21 year-old group still had the highest likelihood of wanting to die, the rates for 22–29 and 30–39 groups were very similar, and the 40 + group’s rates was not too much lower. Considering gender as a main effect, compared to men, odds of wanting to die were 30.4% higher among women across all five countries (*Wald chi-square*(1) = 66.95, p < 0.001, *OR* = 1.30, 95% CI:1.22–1.39).

### Thoughts of Suicide

The 3-way gender * age group * country interaction was not significant (*Wald chi-square*(12) = 18.50, *p* = 0.101). The 2-way age group * country interaction was significant (*Wald chi-square*(12) = 28.69, p = 0.004), and the pattern of results was similar to the pattern reported above, about wanting to die. The 2-way gender * country interaction was likewise significant (*Wald chi-square*(4) = 12.00, p = 0.017). Observing Fig. 2c, women had higher rates of thoughts of suicide in Egypt and Saudi Arabia, but in Algeria, men were more likely (though not significantly so) to report thoughts of suicide. Finally, 2-way gender * age group interaction was likewise significant (*Wald chi-square*(3) = 8.52, p = 0.036), following the same pattern reported for the entire sample, with more women than men reporting thoughts of suicide in the 18–21 group, but no such differences in other groups.

### Suicide Attempt

The 3-way gender * age group * country interaction was not significant (*Wald chi-square*(12) = 11.57, *p* = 0.48). The 2-way gender * country interaction was significant (*Wald chi-square*(4) = 11.96, p = 0.018), with women being more likely to report an attempt than men except in Algeria, where men were as likely to report an attempt as women. The 2-way age group * country interaction was also significant (*Wald chi-square*(12) = 26.68, p = 0.009). Observing Fig. 2d, the rate of attempts was similar among all age groups in Algeria. In other countries, however, 18–21-year-old group was markedly more likely to report an attempt, though there were some differences between countries in other groups. For instance, there were little difference between 22–29, 30–39, and 40 + groups in Jordan and Morocco, and little difference between 30–39 and 40 + groups in Saudi Arabia.

## Discussion

The goal of this study was to examine suicidality among Arabic-speaking adults in the Arab World who visited an online mood screener, and to determine relative prevalence of different levels of suicidality among age groups, gender, and country of residence. Overall, findings suggest that younger adults and women in the Arab World may be more likely to experience suicidality relative to older adults and men. These results are similar to findings from other parts of the world; in the United States, for instance, the highest likelihood for suicidality was likewise observed among the youngest adult age group and the lowest – among the 40s and 50s age group (Han et al., 2015; Kessler et al., 2005).

The rates of suicidality identified in this study were high but not dissimilar to other studies using an online screener in different populations (Leykin et al., 2012; Liu et al., 2014). Individuals experiencing suicidal ideations often seek information and help on the Internet (Lai et al., 2014). Online suicidality screeners may offer a less stigmatizing space for individuals, allowing them to be more open and forthcoming about their suicidality compared to face-to-face clinical interviews with providers (King et al., 2015). Indeed, it should be noted that participants in our study were not a random sample of Arabic speakers residing in the Arab World, but individuals who are interested in browsing for depression and suicide information online.

Suicidality and deaths by suicide in the Arab World are likely to occur due to a variety of factors. For instance, younger women were found to be more likely to report thoughts of suicide, which is similar to studies from elsewhere in the World, which likewise find that women are likely to report attempts (Dardas et al., 2017; Han et al., 2015; Itani et al., 2018; Kessler et al., 2005; Kronfol, 2012; O’Loughlin & Sherwood, 2005). Past studies from the Arab World had also shown that women who have experienced suicidality are likely to be younger (Halabi et al., 2020; Khan & Reza, 1998). Interestingly, however, in this sample, men and women beyond age 21 had similar percentages of thoughts of suicide. There can be several explanations for this unexpected similarity. Individuals in this sample were recruited to a depression and suicide screening website; it is possible that men in this sample were more interested in mental health or more concerned about their own mental health than is typical. Additionally, similarities emerged for thoughts of suicide, and not for actual attempts, which may suggest that men and women living in the Arab world may be similarly likely to consider suicide, but women may be more likely to act on these thoughts.

In most instances, this study found that younger individuals living in the Arab world who use the internet to search for depression information are more likely to experience suicidality than older individuals. Youth may face considerable social pressures that may not be present for older individuals. These pressures may include transition to adulthood as well as social expectations of marriage and employment. However, unemployment among young adults in the Arab World is around 20%, which is the highest worldwide (International Labour Organization 2022). The hypothesis of social pressures may be supported by a study of adolescents in Lebanon who died by suicide that reported these adolescents to be more likely to have experienced child psychological and physical abuse, alcohol use disorders, social anxiety, bullying, and impulsivity (Chahine et al., 2020).

Importantly, this study highlighted differences between countries in the Arab World, suggesting that the Arab World should not be studied as a monolith, but rather as a set of distinct regions. Taking an example of Algeria, for instance, the levels of suicidality in that country were much lower than in the other four countries. Many Arab countries lack mental health legislation and policies (Okasha et al., 2012). In contrast, in Algeria, mental health expenditures by the Health Department represent 7.37% of the total health budget (World Health Organization, 2011). The Algerian state provides psychiatric care that is free of charge to residents, and outpatient care is provided by psychiatric dispensaries distributed across various cities and towns (Benmebarek, 2017; Kacha, 2010). It is therefore possible that, in comparison to the other countries, the openness about mental health and mental health care reduced overall suicidality. Conversely, unlike other countries, Algerians in their 30s were more likely to think of death relative to other age groups. This may suggest a cohort effect among individuals who grew up during rather turbulent economic and political times in the 1990s and early 2000s; this pattern of findings warrants further research.

A significant advantage of using an online survey was that it allowed access to hard-to-reach populations, especially participants separated by geographical distances in the Arab World. It also allowed participants to respond in a confidential manner. Despite the advantages of online survey, the study had several limitations. This study relied on Google Ads for recruitment; as such, participants were a self-selected group that was searching online for depression information, which may limit the generalizability of findings. Similarly, findings may not generalize to individuals from lowest socioeconomic strata, individuals without access to the internet, or individuals whose mental health literacy is poor. Although the study recruited a very large sample, it is not a representative sample of the Arab population, and especially not every country in the Arab World, given that some countries were better represented than others in the sample. The study was conducted in Modern Standard Arabic, and though it is understandable by the vast majority of Arabic speakers in the Arab world, it is possible that nuances used in local dialects were not represented.

Suicidality in the Arab World remains understudied. This study explored suicidality and its demographic characteristics, across and between several Arab countries using an online sample. Generally, findings indicated that pattern of suicidality in the Arab World is not dissimilar to patterns elsewhere, in that in our sample of individuals living in the Arab world who use the Internet to search for depression information younger individuals are likewise more likely to experience suicidality than older individuals, and women are more likely to experience suicidality relative to men. However, findings also revealed differences in prevalence between countries, which highlights the need to identify country- and region-level differences in suicidality risk factors for better prevention and treatment efforts.
